# Mechanical Performance of Portland Cement, Coarse Silica Fume, and Limestone (PC-SF-LS) Ternary Portland Cements

**DOI:** 10.3390/ma15082933

**Published:** 2022-04-18

**Authors:** Miguel Ángel Sanjuán, Esperanza Menéndez, Hairon Recino

**Affiliations:** 1 Spanish Institute of Cement and its Applications (IECA), C/José Abascal, 53, 28003 Madrid, Spain; 2 The Eduardo Torroja Institute for Construction Science (Spanish National Research Council, CSIC), C/Serrano Galvache, 4, 28033 Madrid, Spain; emm@ietcc.csic.es (E.M.); h.recino@ietcc.csic.es (H.R.)

**Keywords:** ternary cements, coarse silica fume, limestone, supplementary cementitious materials, multicomponent binders, eco-friendly construction materials, properties of cementitious materials

## Abstract

Ternary Portland cements composed of coarse silica fume (SF), limestone (LS), and Portland cement (PC) can afford some environmental advantages by reducing the clinker content in Portland cements. These cements will help to reduce the clinker factor target from 0.78 to 0.60 by 2050 with the aim to be climate neutral. Silica fume (SF) possesses pozzolanic properties that enhance mechanical strength and durability. By contrast, limestone powder has three main outcomes, i.e., filler, dilution, and chemical effects. The first reduces porosity and refines the microstructure of mortars and concretes. The second decreases the amount of hydration products and increases the porosity; the third one promotes the appearance of carboaluminates and reduces porosity. This paper covers the mechanical properties of Portland cement-limestone-coarse silica fume ternary cements, and its synergetic mechanism. Compressive and flexural strength of mortar at 2, 7, 14 and 28 days was performed. Coarse silica fume has a minor contribution on the nucleation effect compared to ground limestone at early ages. The nucleation and filler effects, at early ages, are less pronounced in coarse and very fine limestone powder. The highest compressive strength at 28 days is reached with the lowest content of coarse silica fume (3%). Mortar mixes made with a high level of limestone presented a delay in the compressive strength development.

## 1. Introduction

The European Union (EU) and all 27 EU Member States are committed to a binding target of a 55% reduction in greenhouse gas emissions by 2030 compared to 1990 levels [1]. Member States shall ensure that the industrial sectors adopt a balanced approach by considering the available measures to address the Climate Change issue. The Industrial Strategy for Europe [2] highlights that the main challenge that EU industries will be facing during the next years is carbon neutrality. Accordingly, the Portland cement industry is dealing with complex operational challenges, i.e., their modernization and decarbonization are strategic issues [3].

The Portland cement industry is a contributing sector to carbon dioxide emissions mainly due to the calcination process and fuel combustion [4], and the production and use of ternary cements is a lever to achieve the 2030 target of reducing carbon dioxide emissions to 55% below 1990 levels and, furthermore, climate neutrality by 2050, i.e., net-zero carbon dioxide emissions [1].

An ambitious target of lowering the worldwide clinker factor from 0.78 to 0.60 has been set for 2050 [5]. This entails drawing up a procedure based on current scientific knowledge to develop alternatives to the conventional Portland cement types manufactured with a high proportion of clinker.

Ternary cements may be defined as binders composed of Portland cement clinker and two other components, such as silica fume and limestone, which could be blended at the cement mill.

Different studies on ternary cements have been performed combining silica fume with coal fly ash [6], silica fume with ground granulated blast-furnace slag [7], limestone with ground granulated blast-furnace slag [8], coal ash with rice husk ash [9], and coal fly ash with calcium carbonate [10]. Then, ternary cements were widely used to reduce carbon dioxide emission. Conventional Portland cement constituents are mainly obtained from industrial wastes, such as coal fly ash and ground granulated blast-furnace slag. Nevertheless, these industrial wastes have become limited because coal thermal plants are likely to run at a low plant load factor in the future to accommodate renewable energy into the grid and also due to the advanced technology of the steel industry and the stricter environmental protection policy.

Ternary cements manufactured with limestone and a third constituent, such as silica fume, is a promising approach due to its availability and lower environmental impact.

Ground limestone has been widely used in common Portland cements, and can influence the properties of Portland cement with limestone by dilution, filler, nucleation, and chemical effects, mainly depending on its particle size distribution (PSD) and amount. The limestone particle size influences the hydration characteristics, i.e., fine limestone powder has a great effect for physical acceleration of the Portland cement hydration at early ages, because this fine powder lowers the activation energy of the Portland cement hydration reaction and enhances the dissolution of Portland cement and additions [11]. By contrast, coarse limestone powder has a less pronounced effect [12].

The dilution effect reduces the mechanical performance of cement-based materials at later ages [13,14].

Limestone powder fine particles acting as fillers can improve the packing of Portland cement and therefore can reduce the interstitial capillary pores. A good concrete durability is primarily achieved by providing low capillary porosity and improved aggregate–paste interfacial transition zone (ITZ) because of low water/cement ratios, which is the weakest area of cement-based materials’ microstructure. Such ITZ contains large portlandite and ettringite crystals [13]. In addition, portlandite presents an oriented growth. Limestone, among other fine inert materials, can be used for improving the ITZ microstructure. Kepniak et al. [13] reported a change in the transition zone microstructure, which is strengthened leading to increased compressive strength and durability of concrete.

Packing density increases at low levels of limestone powder, less than 10–15% [15,16]. A noticeable reduction in the interparticle spacing enhances hardening and early age mechanical properties. Furthermore, it reduces setting time.

Knop and Peled [17] reported an increase in packing density from 0.53 to 0.58 when the limestone powder content increases from 5 to 35%. Thus, ground limestone can be considered as low-cost filler for packing optimization to design special concretes, such as self-compacting concrete [18].

Moon et al. [19] reported that cements with 15% limestone increase the mechanical performance of plain cements due to the nucleation and filler effects, which promote Portland cement hydration and porosity decreases. By contrast, the use of 25–35% of limestone as replacement for plain Portland cement presented lower concrete compressive strength than the reference concrete, which can be justified by the dilution effect, which inhibits Portland cement hydration.

Furthermore, under portlandite saturation conditions, aluminum reacts with limestone powder. Then, limestone in blended cements reacts with aluminate (mainly tricalcium aluminate, C_3_A) phases and portlandite, Ca(OH)_2_, at early hydration times in calcite-containing Portland cements to form hemicarboaluminate. At a late age, the conversion of hemicarboaluminate into monocarboaluminate proceeds [20].

Hemicarboaluminate (1.98 g/cm^3^) and monocarboaluminate (2.17 g/cm^3^) have lower density compared to C-S-H gel (2.22–2.33 g/cm^3^), C-A-S-H gel (1.5–2.4 g/cm^3^), portlandite (2.20 g/cm^3^), and monosulphate (2.02 g/cm^3^) [21], which is beneficial for the pore space filling process.

The extent of limestone powder that can react with the tricalcium aluminate, C_3_A, is quite small, about 2–6%, due to the low content of C_3_A in Portland cements, particularly in sulphate-resistant cements. Accordingly, to increase the limestone powder reactivity and the replacement level of Portland cement, it is recommended to increase the content of aluminous phases [22,23]. Dhandapani et al. [24] reviewed a large range of ternary binders involving limestone. They concluded that carboaluminates generation cannot be considered as the primary reason for characteristics enhancement in these limestone ternary binders. Furthermore, they highlighted that limestone varies the early age hydration kinetic of blended cements in a different manner depending on the particle size distribution (PSD), nature, and content of limestone.

Fillers are inert or less reactive materials used to replace cementitious materials in Portland cement. Typical fillers are limestone powder and ground quartz. Furthermore, unreactive parts of supplementary cementitious materials or cement additions, such as quartz, mullite, and so on, act as fillers in blended cements. They lead to a reduction in the reactive constituents of Portland cement, i.e., this results in dilution. Nevertheless, proper use of the water to binder ratio (w/b) can counteract dilution effect at later ages [25]. In addition, limestone powder can improve the degree of hydration of clinker phases [26].

Silica fume is a byproduct in the silicon and ferrosilicon industry, generated in the carbothermic reduction of high-purity quartz with coal or coke in electric arc furnaces at temperatures up to 2000 °C. In this thermal process, SiO_2_ vapors oxidize and condense in the low-temperature zone to very fine spherical particles consisting mainly of non-crystalline silica (about 85–95%) and small contents of iron, magnesium, and alkali oxides.

Utilization of industrial by-products in construction materials has become an attractive alternative to disposal. In addition, silica fume is a very effective constituent of high-strength and high-performance concrete [27] due to its very reactive pozzolanic characteristics. Silica fume plays three roles in cement-based materials. First, the amorphous silicon dioxide reacts with free-lime; second, matrix densification and pore-size refinement are produced; and third, cement paste–aggregate and paste–steel reinforcement interfacial refinement occurred [28].

Mazloom et al. [29] investigated the influence of silica fume (0–15%) on the compressive strength of high-performance concrete. They found that silica fume concrete had a compressive strength 21% higher than the reference one at 28 days; however, the compressive strength development of silica fume concrete was negligible after 90 days. By contrast, they observed an increase of compressive strength of 26% and 14% in the control concrete after one year compared to the values recorded at 28 and 90 days, respectively. Wild et al. [30] attributed this fact to the formation of an inhibiting layer of reaction products in the silica fume concrete, preventing further reaction of non-crystalline silica with calcium hydroxide beyond 90 days.

Wong and Razak [31] studied the compressive strength of silica fume concrete with water-to-binder ratios of 0.27, 0.30, and 0.33. They found that silica fume did not provide an instant compressive strength enhancement; conversely, they achieved higher compressive strength than the control concrete from seven days onwards. What is more, compressive strength loss at early ages, which was proportional to the Portland cement replacement, was due to the dilution effect. Bentur et al. [32] reported that silica fume eliminates the weak interfacial region by strengthening the cement paste-aggregate bond and forming a more homogenous and less porous microstructure in the mentioned interfacial zone. Finally, Bhanja and Sengupta [33] observed a pronounced effect on flexural strength of silica fume in comparison with splitting tensile strength.

The silica fume fineness according to EN 13263-1:2005+A1:2009 “Silica fume for concrete—Part 1: Definitions, requirements, and conformity criteria” [34] should have a specific surface between 15,000–35,000 m^2^/kg, and from 300 m^2^/kg to 400 m^2^/kg according to EN 16622:2015 “Silica-calcium fume for concrete—Definitions, requirements, and conformity criteria” [35]. However, in the current context of Climate Change mitigation and in order to lower the worldwide clinker factor from 0.78 to 0.60 by 2050 [5], new cement constituents should be incorporated in the new cement standards. Accordingly, the utilization of coarse silica fume can be considered as a potential new cement constituent with lower properties than the conventional silica fume and silica-calcium fume. The assessment of the mechanical performance of ternary mixes made with coarse silica fume, limestone, and Portland clinker is the aim of the present research program. The mechanical performance should be considered in the decision-making process regarding the use of these new ternary cements. Hence, this paper covers the mechanical properties of clinker-limestone-coarse silica fume ternary cements and its synergic effect. It deals with the effect of limestone and coarse silica fume on the compressive strength and flexural strength of mortar.

## 2. Materials and Methods

### 2.1. Raw Materials

In this study, Portland cement (PC), coarse silica fume (SF) and limestone (LS) were used as raw materials to prepare ternary cements. Portland cement of grade 42.5 (CEM I 42.5 R according to the European standard EN 197-1) and the limestone were obtained from LafargeHolcim España Cement Co., Villaluenga de la Sagra, Toledo, Spain. A coarse silica fume (SiO_2_) was provided by Ferroatlántica, Sada, Spain. Their chemical is given in Table 1. A complete elemental chemical analysis of the samples is performed. Most of the elements were analyzed using the molten pearl X-ray fluorescence technique, with a wavelength scattering X-ray spectrometer, Bruker’s S8 Tiger. Loss on ignition (LOI) and sulfate content determination are described in the European standard EN 196-2 [36]. The alkali content (Na^+^ and K^+^) in cement, limestone, and coarse silica fume was determined by inductively coupled plasma optical emission spectrometry (ICP-OES), with a Varian model 725-ES equipment. The mineralogical phases present in Portland cement, coarse silica fume, and limestone are given in Table 2.

Limestone was ground until the required degree of fineness was achieved. The three materials produced from the same limestone but of widely different fineness were obtained and coded as **10** (8001 cm^2^/g), **20** (25,857 cm^2^/g), and **50** (25,954 cm^2^/g). Grinding time was 10, 20, or 50 min, respectively. Limestones 20 and 50 had similar fineness.

The Blaine air-permeability apparatus is used to determine the specific surface of the cement, based in the ASTM C204 standard. In the rest of the raw materials, coarse silica fume, and limestone, the specific surface is determined by the sieving method with an ALPINE E200LS Sieve Shaker. On the other hand, the density of all raw materials is determined by the Le Chatelier Flask method described in ASTM C188 standard.

**Figure 1:**
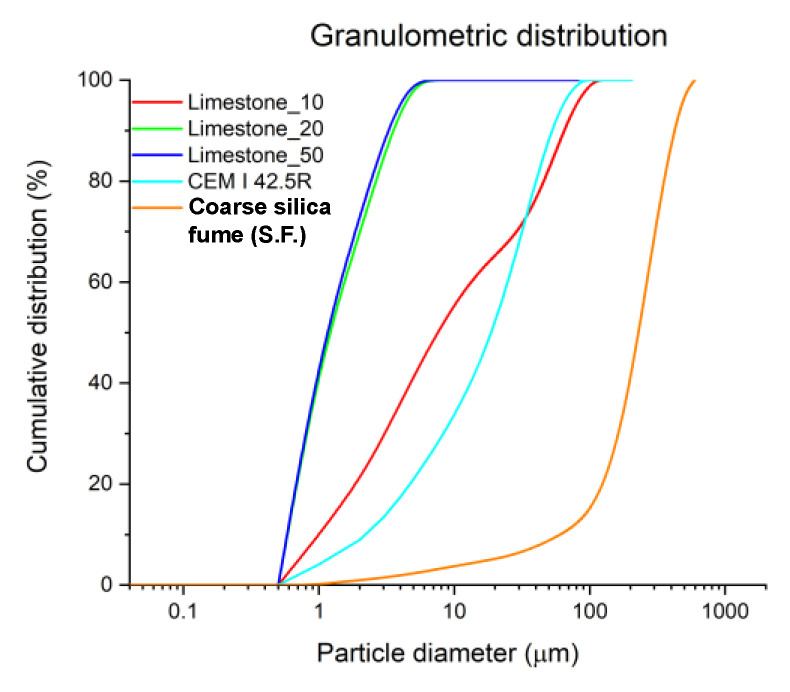
Particle size distribution (PSD) of the materials used to manufacture ternary cements.

Next, in Table 3, the density and specific surface values of the raw materials studied are shown. The determination of the granulometric fractions of the raw material samples was carried out with a Malvern laser diffractometer model Mastersizer2000. In Figure 1, the granulometric distribution or particle size distribution (PSD) of the analyzed samples is shown. The coarsest material is the coarse silica fume, while the 10-limestone fineness is most similar to that of the Portland cement. The finest materials are the 20 and 50 limestones. Furthermore, both of them present almost the same particle size distribution (PSD).

### 2.2. Ternary Cement Mix Design

The ternary cements were composed of Portland cement, coarse silica fume and limestone. The amount of coarse silica fume was 3%, 5%, and 7%. Limestone was ground and three different fineness were obtained. The average limestone (10-limestone) was mixed in percentages of 10%, 15%, and 20%, whereas the finest limestones were added only in a proportion of 5%. Table 4 shows the mix design defined to manufacture ternary cements.

### 2.3. Mechanical Strength

The mechanical assessment was performed at 1, 7, 14, and 28 days following the compressive strength testing method defined in the European standard EN 196-1 [37].

Flexural strength test result for each type of mortar is calculated as the arithmetic mean of three individual mortar results, which were obtained from a flexural strength test measurement made on a set of three mortar prisms. Conversely, compressive strength test results for each type of mortar are calculated as the arithmetic mean of six individual mortar results, which were achieved from a compressive strength test measurement made on a set of six mortar specimens. Such individual test results cannot vary by more than ±10% from the mean. A result outside this limit will be discarded and a new arithmetic mean of the five remaining results will be considered. In the case of two results outside this limit, the set of results will be discarded. Therefore, measurement error of the test results, summarized in terms of precision, is lower than 2% in agreement with the EN 196-1 [37]. In this paper, a difference between two values higher than 1.5 MPa is considered to be statistically significant.

According to Huang and Shen [38], the 28-days compressive strength standard deviation of Portland cement mortars ranges from 0.2 to 1.2 MPa. Furthermore, the precision of mortar test was not sensitive to cement type [39].

## 3. Results and Discussion

### 3.1. Flexural Strength

**Figure 2:**
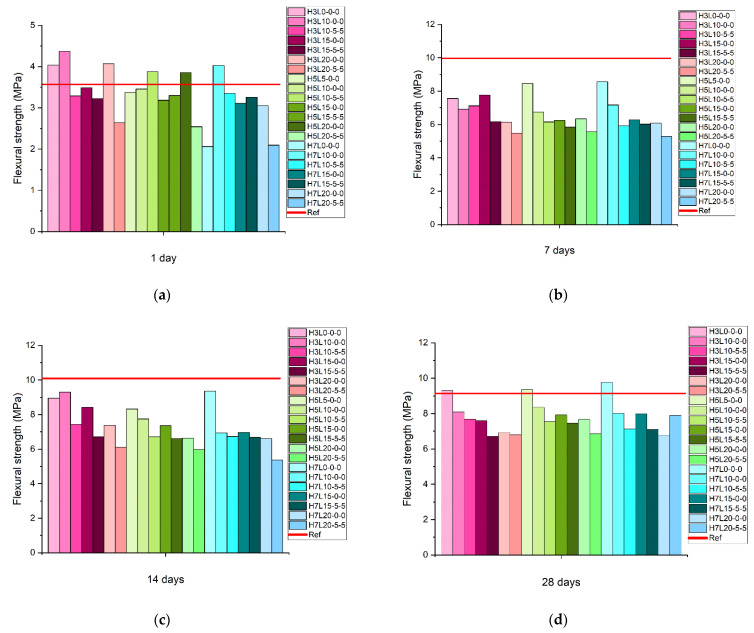
Flexural strength of mortars made with limestone and three contents of coarse silica fume (3%, 5%, and 7%) at: (**a**) 1 day, (**b**) 7 days, (**c**) 14 days, and (**d**) 28 days.

Figure 2 shows the flexural strength results of mortars made with limestone and different contents of coarse silica fume (3%, 5%, and 7%) at 1, 7, 14, and 28 days. The highest flexural strength at 1 day is reached with the lowest coarse silica fume content (3%) and 10% of the average limestone (code 10). It was reported that ground limestone finer than the Portland cement has a positive effect on the early age hydration. This limestone is slightly finer than 10 μm. Therefore, this fine limestone powder has a great effect for physical acceleration of the Portland cement hydration at early ages by lowering the activation energy of said reaction. In addition, it enhances the dissolution of the Portland cement constituents [11]. This effect is less pronounced in coarse limestone powder [12]. In addition, very fine limestone powder (H3L20-5-5) is less effective than the average limestone powder (H3L20-0-0). As expected, the highest limestone content (40%) provides the lowest flexural strength at 1 day (H3L20-5-5, H5L20-5-5 and H7L20-5-5). In particular, a low content of coarse silica fume (3%) showed better results in terms of the mechanical strength. This can be attributed to the dilution effect, which reduces the mechanical performance of mortars and concretes [13,14].

Unexpectedly, the reference mortar with 7% of coarse silica fume gave an anomalous result at 1 day. However, at 7, 14, and 28 days, all the mortars with 7% of coarse silica fume followed a clear trend, i.e., the higher the limestone content, the lower the flexural strength. Blended mortar’s flexural strength at 7 and 14 days was lower than results obtained with the reference mortar without any addition (line red in Figure 2b,c). Accordingly, the dilution effect decreases the mechanical performance of cement-based materials at later ages [13,14]. Finally, the flexural strength results at 28 days followed the same trend for the mortars made with 3%, 5%, and 7% of coarse silica fume.

### 3.2. Compressive Strength

**Figure 3:**
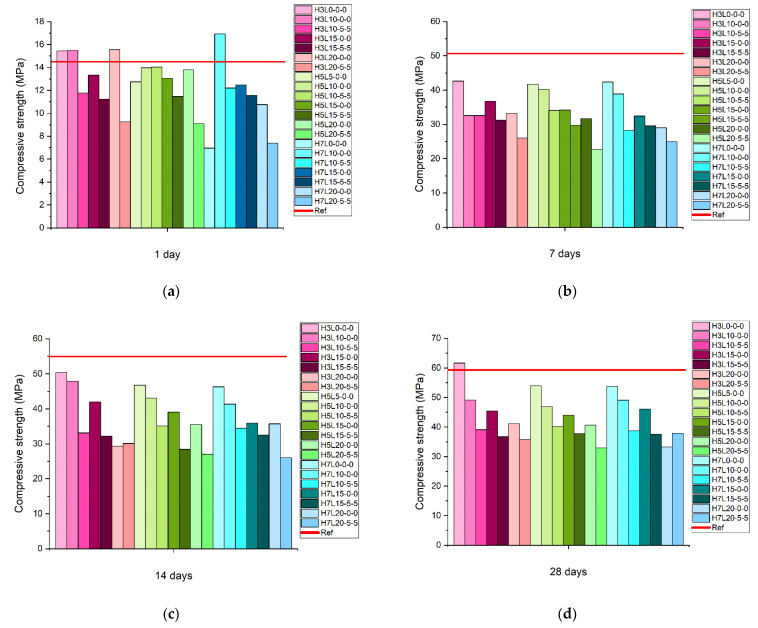
Compressive strength of mortars made with limestone and three contents of coarse silica fume (3%, 5% and 7%) at: (**a**) 1 day, (**b**) 7 days, (**c**) 14 days, and (**d**) 28 days.

Figure 3 shows the compressive strength results at 1, 7, 14, and 28 days of mortars made with limestone and 3%, 5%, and 7% of coarse silica fume. The ground limestone with an average particle size of about 10 μm has a positive effect on the early age hydration (1 day). By contrast, the finest limestone with a mean particle size of 2 μm does not present a beneficial effect. On the contrary, Moon et al. [19] observed that limestone with an average size of 1 μm provided higher compressive strength and lower porosity than limestone with an average size of 10 μm.

This fact suggests that limestone fineness in the presence of coarse silica fume has a more dominant impact on early strength than hydration characteristics. It is also raised that the finest particle size positively influences the nucleation effect; however, the packing process can be enhanced more effectively with an adequate grinding fineness, i.e., neither coarse-grained nor too fine. Accordingly, the Portland cement fineness is recommended.

At 7 and 14 days, the compressive strength results of mortars made with limestone and 3%, 5%, and 7% of coarse silica fume were much lower than the compressive strength achieved by the reference mortar made without additions. The significant reduction in compressive strength is due to the dilution effect created by the inclusion of a substantial content of limestone. The effect of the coarse silica fume content is negligible for all the proportions studied (3%, 5% and 7%) at 7 and 14 days. Coarse silica fume mortars (H3L0-0-0, H5L0-0-0 and H7L0-0-0) showed a compressive strength of about 40 MPa and 50 MPa, while the reference mortar has 50 MPa and 55 MPa at 7 and 14 days, respectively. Therefore, in this period of time, clinker content was found to have the highest impact on the strength development.

Eventually, compressive strength data at 28 days followed the same trend regardless of the coarse silica fume content in the mortars (3, 5, or 7%). Nevertheless, the lower the coarse silica fume content, the highest the compressive strength at 28 days.

Furthermore, the highest compressive strength result at 28 days was obtained with the coarse silica fume mortar with the lowest amount of coarse silica fume (3%) and without limestone (H3L0-0-0). This value was slightly higher (62 MPa) than the compressive strength of the reference mortar (59 MPa). On the contrary, in the other cases, the compressive strength is much lower than the reference mortar. The compressive strength data at 28 days decreases sharply as limestone increases, regardless of the limestone fineness. Thus, the dilution effect plays a key role. Gołaszewski et al. [40] reported similar results. They suggested that the loss in compressive strength at higher replacement levels is independent of limestone particle size. In addition, one concern with the utilization of ultrafine limestone less than 2 μm was the proper dispersion of the limestone particles during the blending process. It should be mentioned that the precision of mortar test is not sensitive to cement type [38] and the 28-days compressive strength standard deviation of Portland cement mortars ranges from 0.2 to 1.2 MPa.

The pozzolanic reaction of the coarse silica fume can partially compensate the dilution effect caused by limestone replacement. This effect has been found in coal fly ash—ground limestone mixes [41,42,43,44]. Furthermore, in ternary cements, the mechanical strength development is governed by the total solid volume of hydrates and C-S-H gel, which is increased due to the pozzolanic reaction [45]. In addition, the presence of calcite in lime—pozzolan systems enhances the reactivity of the pozzolanic materials [46] as result of the synergistic contribution of the interaction of the calcite with the aluminosilicate.

It has been reported that packing density increases with the decrease of the addition content (<15%) of ground limestone [16,47]. In addition, fine limestone particles as fillers can improve the packing density of the ternary cement, and in turn, diminishes the interstitial capillary pores [48]. Therefore, a critical factor on the limestone content is the dilution effect, which decreases the compressive strength at 28 days [49,50,51]. It is well known that this negative effect is contrary to other Portland cement constituents, such as coal fly ash, natural pozzolan, and ground granulated blast-furnace slag, wherein their use in cement-based materials reduces early compressive strength and contributes to later age mechanical properties [52].

In this paper, coarse silica fume (45.1 m^2^/kg) is used. Coarse silica fume exhibits physical properties that significantly differ from those at a smaller scale, such as silica fume (15,000–35,000 m^2^/kg) conforming to EN 13263-1:2005+A1:2009 [34] or silica-calcium fume (300 m^2^/kg to 400 m^2^/kg) conforming to EN 16622:2015 [35]. New additions should be assessed to enhance the Climate Change mitigation. There is a target of lowering the worldwide clinker factor from 0.78 to 0.60 by 2050 [5]. To this end, the viability of byproducts and industrial wastes, such as cement constituents, should be reviewed.

In conclusion, limestone and silica fume combinations were found to give a promising mechanical performance, accommodating up to 30% ground limestone with an acceptable loss in compressive strength in blended mortars. Accordingly, these new ternary cements hold promising opportunities for increasing ground limestone replacement levels. Particle size distribution (PSD) of the Portland cement constituents has a strong linkage with the compressive strength development [53]. Then, by adjusting the fineness of a ternary cement, a positive effect on the early age hydration can be achieved. Furthermore, different particle size distribution (PSD) could affect the benefits of limestone, i.e., filler, dilution, and chemical effects.

## 4. Conclusions

From the results of the experimental and analytical study presented in this paper, the following conclusions are reached:

1. The coarse silica fume has a less impact on the nucleation effect than the ground limestone at early ages (1 day).
2. The nucleation and filler effects at early ages are less pronounced in coarse and very fine limestone powder. Furthermore, limestone with a fineness similar to that of cement is more effective, with regard to the mechanical strength, than the finest limestone.
3. The highest compressive strength at 28 days is reached with the lowest silica fume content (3%). Accordingly, the coarse silica fume enhances compressive strength due to the pozzolanic reaction at low levels of replacement. By contrast, higher substitution contents provide a slight dilution effect, which will reduce the mechanical performance of cement-based materials.
4. Apparently, the loss in compressive strength at high limestone replacement levels is independent of its particle size. Nevertheless, a concern of the blending process of the ultrafine limestone is the proper dispersion of the particles.

These findings can be a useful tool for material and civil engineers in designing low-carbon concrete products made with these ternary cements.

## Figures and Tables

**Table 1:** Chemical composition of the CEM I 42.5 R, coarse silica fume, and limestone.

Chemical Composition (%)	CEM I 42.5 R	Coarse Silica Fume	Limestone
CaO	63.02	0.39	46.26
SiO_2_	19.80	96.09	3.35
Al_2_O_3_	4.55	0.14	1.57
SO_3_	3.16	0.09	0.07
Fe_2_O_3_	2.67	0.07	0.37
MgO	1.93	0.12	0.30
K_2_O	0.89	0.42	0.22
Na_2_O	0.29	0.19	0.05
SrO	0.07		0.02
Cl^−^	0.03		
TiO_2_	0.22		0.06
P_2_O_5_	0.15		
MnO			0.01
LOI ^1^	3.21	2.50	47.70
Na_2_O_eq_^1^	0.88	0.47	0.20

^1^ LOI: Loss on ignition; Na_2_O_eq_ = Na_2_O + 0.658 K_2_O.

**Table 2:** Mineralogical phases of the CEM I 42.5 R, coarse silica fume, and limestone.

CEM I 42.5 R		Coarse Silica Fume		Limestone	
C_3_S	66.48%	Amorphous	99.12%	Calcite	93.25%
Amorphous	11.36%	Quartz	0.88%	Quartz	2.04%
C_4_AF	6.97%			Amorphous	4.74%
Gypsum	6.38%				
C_2_S	5.99%				
C_3_A	2.44%				
Calcite	0.38%				

**Table 3:** Density and specific surface area (Blaine) of the raw materials used to manufacture ternary cements.

Raw Materials	Density (g/cm^3^)	Specific Surface Area, Blaine (cm^2^/g)
CEM I 42.5R	3.16	3522
Limestone 10	2.77	8001
Limestone 20	2.64	25,857
Limestone 50	2.77	25,944
Coarse silica fume	1.85	451

**Table 4:** Raw materials and mix design to manufacture ternary cements, %.

Code	CEM I	Coarse Silica Fume	Limestone 10	Limestone 20	Limestone 50
Reference ^1^	100	0	0	0	0
H3L0-0-0	97	3	0	0	0
H3L10-0-0	87		10	0	0
H3L10-5-5	77		10	5	5
H3L15-0-0	82		15	0	0
H3L-15-5-5	72		15	5	5
H3L20-0-0	77		20	0	0
H3L20-5-5	67		20	5	5
H5L5-0-0	95	5	0	0	0
H5-10-0-0	85		10	0	0
H5L10-5-5	75		10	5	5
H5L15-0-0	80		15	0	0
H5L15-5-5	70		15	5	5
H5L20-0-0	75		20	0	0
H5L20-5-5	65		20	5	5
H7L0-0-0	93	7	0	0	0
H7L10-0-0	83		10	0	0
H7L10-5-5	73		10	5	5
H7L15-0-0	78		15	0	0
H7L15-5-5	68		15	5	5
H7L20-0-0	73		20	0	0
H7L20-5-5	63		20	5	5

^1^ The reference Portland cement is a CEM I 42.5 R according to the European standard EN 197-1.

## Data Availability

Not applicable.
